# Isolation and Identification of Optochin-Resistant Viridans Group Streptococci from the Sputum Samples of Adult Patients in Jakarta, Indonesia

**DOI:** 10.1155/2021/6646925

**Published:** 2021-07-14

**Authors:** Wisiva Tofriska Paramaiswari, Nurma Sumar Sidik, Miftahuddin Majid Khoeri, Wisnu Tafroji, Wahyu Finasari Said, Dodi Safari

**Affiliations:** ^1^Eijkman Institute for Molecular Biology, Jakarta, Indonesia; ^2^Department of Biochemistry, Bogor Agricultural University, Bogor, Indonesia; ^3^Department of Clinical Pathology, Faculty of Medicine Universitas Indonesia/Cipto Mangunkusumo Hospital, Jakarta, Indonesia

## Abstract

**Aim:**

To investigate optochin-resistant viridans group streptococci (VGS) strains isolated from the sputum sample of adult patients with different clinical symptoms.

**Materials and Methods:**

Optochin-resistant VGS isolates were identified by matrix-assisted laser desorption ionization time of flight mass spectrometry (MALDI-TOF MS). *recA* sequencing was used to confirm identified isolates at the genus level by MALDI-TOF MS. *Finding*. We identified 79% of tested isolates (148/187) at the species-level identification using the MALDI-TOF MS tool. We identified that the most common species isolated from sputum specimens were *S. oralis* (44.9%) followed by *S. mitis* (25.7%), *S. infantis* (9.1%), *S. parasanguinis* (7.5%), *S. peroris* (3.7%), *S. anginosus* (2.7%), and *S. sanguinis* (2.1%). *Discussion.* The *S. oralis* strains were majority of optochin-resistant VGS isolates obtained from sputum of adult patients in Jakarta, Indonesia. MALDI-TOF MS showed potential for the rapid identification tool to identify optochin-resistant VGS isolates. Although there were discrepancies in identifying isolates at the genus/species level, the performance could be improved by expanding its database.

## 1. Introduction

The high-level similarities between *Streptococcus pneumoniae,* a human pathogen, and viridans group Streptococci (VGS), particularly within the nonpneumococcal mitis group including *Streptococcus mitis*, *Streptococcus oralis*, and *Streptococcus pseudopneumoniae*, often cause difficulties in species discrimination [1, 2]. In clinical laboratory testing, conventional tests such as optochin sensitivity and bile solubility are still applied as key identifications for *S. pneumoniae* isolates [3]. However, some *S. pneumoniae* isolates were reported as optochin resistant in different geographical regions [4].

The VGS, a group of catalase-negative, Gram-positive Cocci, are a heterogeneous group of bacterium and considered to be normal flora of the oropharyngeal, urogenital, and gastrointestinal microbiota [5]. Classification of VGS has been challenging due to variability and overlap of their microbial characteristics [6]. This bacteria group includes a diverse range of organisms within the genus *Streptococcus* and can be characterized by green coloration on a blood agar plate [7]. Currently, VGS are classified into six major groups: the *S. mutans* group, *S. salivarius* group, *S. anginosus* group, *S. mitis* group, *S. sanguinis* group, and *S. bovis* group [5, 8]. The pathogenicity of VGS ranges from opportunistic pathogens causing mild disease such as *S. mutans* that strongly correlates with dental caries development, and *S. mitis*, *S. oralis*, and *S. sanguinis* are taking roles in infective endocarditis [8].

Specific and accurate species-level identification of VGS is one of the important factors in patient clinical management and is also important for understanding their pathogenicity and virulence [1, 9]. Matrix-assisted laser desorption ionization time of flight mass spectrometry (MALDI-TOF MS) has become an indispensable tool for clinical microbiology laboratories and shown to be a potential alternative for organism identification with a rapid and cost-saving method for VGS identification [2, 5, 9]. Previously, we reported that thirteen *S. pneumoniae* (pneumococcus) strains were susceptible to the optochin test and one hundred and eighty-nine of alpha haemolytic nonpneumococcus strains were resistant to the optochin test from the sputum of adult patients with nonspecific clinical symptoms in Jakarta, Indonesia [10]. In this study, we investigate further nonpneumococcus strains from adult patients for optochin-resistant VGS identification by the MALDI-TOF MS.

## 2. Methods

### 2.1. *Streptococcus* Group Collection

The *Streptococcus* group isolates were archived isolates obtained from sputum samples of adult patients with different clinical symptoms aged 18–87 years in Jakarta, Indonesia [10]. The patient clinical symptoms are tuberculosis (*n* = 51), community acquired pneumonia/healthcare-associated pneumonia (*n* = 17), SIDA/AIDS (=10), diabetes mellitus (*n* = 6), pneumonia sepsis (*n* = 3), pneumonia (*n* = 2), other symptoms (*n* = 66), and missing data (*n* = 32). The sputum samples were inoculated onto blood agar plates supplemented with 5 mg/L of gentamicin and were incubated at 37°C in 5% CO_2_ for 18–24 h. All isolates that are alpha-hemolytic, resistant to optochin disk (ethylhydrocupreine hydrochloride), and insoluble in bile were included in this study [10].

### 2.2. Sample Preparation

All isolates were subcultured on a tryptone soya agar plate with 5% sheep blood and then incubated overnight at 37°C with 5% CO_2_ [10]. A single colony of overnight pure growth bacteria was spotted to the MSP 96 ground plate (Bruker Daltonik, Germany) using a sterile toothpick and air dried in room temperature for approximately 5 minutes as a direct method sample preparation. The dried spots were then mixed with 1 *μ*L matrix (saturated solution of *α-cyano-4-hydroxycinnamic acid/*HCCA in 50% acetonitrile and 2.5% *trifluoroacetic acid* (TFA)). The solution was air dried in room temperature for approximately 10 mins. Standard protein extraction method was used to confirm the isolates with MALDI-TOF identification score <2.000 [11]. A 2.0 McFarland of bacterial suspension was made in 300 *μ*L of water and then mixed with 900 *μ*L of ethanol. The suspension was homogenised and centrifuged at 20000 × g for 2 minutes. The supernatant was removed, and the pellet was dried at 55^o^C for 30 minutes. The dried pellet was resuspended in 50 *μ*L of acetonitrile followed by centrifugation at 20000 × g for 2 minutes. A 1 *μ*L supernatant was spotted to the ground plate and air dried for 10 mins in room temperature. Then, 1 *μ*L of matrix was added to the same spot as in the direct colony method as described above.

### 2.3. MALDI-TOF-MS-Based Identification

The isolates were identified using Microflex MALDI-TOF (Bruker Daltonik, Germany) and flexControl version 3.4 software as previously described [2, 12]. Isolate identification was performed from spectrum acquisition was conducted in the positive linear mode with laser frequency at 60 Hz. Mass range started at 2.000–20.000 Da. Each voltage from ion source 1 and ion source 2 was set at 20 kV and 18.5 kV. Bacterial test standard protein was included in every test as instrument calibration. Automatic identification started after the spectra result was moved to Biotyper RTC software. The identification criteria were based on the similarity level, shown by the logarithmic score of isolates and database spectra prior to instructions by the manufacturer as follows: score <1.700 indicated isolates were not reliably identified; isolates with score 1.700–1.999 indicated identification accuracy up to the genus level; and isolates with score ≥2.000–3.000 indicated isolates accurately identified up to the species level. Mass spectra analysis was conducted using flexAnalysis software. All obtained spectra were saved in flexControl before undergoing the calibration, smoothing, and baseline subtraction process on flexAnalysis MBT-Standard, prior to the manufacturer's recommendation.

### 2.4. *recA* Sequencing Identification

The *recA* sequencing tool was used to confirm all identified isolates at the genus level by MALDI-TOF MS [13]. We performed *recA* gene amplification and sequencing using forward primer [5′-GCCTTYATCGATGCBCARCA-3′] and reverse primer [5′-GTTTCCGGRTTDCCRAACAT-3′] with the GoTaq Green Mastermix [13]. The obtained sequences were compared to the *rec*A gene sequences database in NCBI GenBank and analysed using BLAST alignment (http://www.ncbi.nlm.nih.gov/blast) and MEGA-6 software. The obtained sequences with similarity ≥96% on published sequences in GenBank were assigned as cutoff for species identification.

## 3. Results

In this study, MALDI-TOF MS identified 79% (148/187) isolates with score value ranging from ≥2.000–3.000, indicating the highly probable species identification result. The majority identified species was *S. oralis* (50.7%), followed by *S. mitis* (31.1%), *S. parasanguinis* (9.5%), *S. anginosus* (3.4%), *S. sanguinis* (2.7%), *S. peroris* (2.0%), and *S. pseudopneumoniae* (0.7%) (Table 1). Meanwhile, we observed that 21% (39/187) of optochin-resistant VGS isolates were identified at the genus level (ID score value: 1.700–1.999) with majority isolates identified as *S. oralis* (38.5%) followed by *S. mitis* (23.1%), *S. peroris* (20.5%), *S. pneumoniae* (12.8%), *S. parasanguinis* (2.6%), and *S. infantis* (2.6%) (Table 2). The identification scores obtained from the isolates extracted using the standard protein extraction method showed no significant difference with those using the direct colony method with the score ranging from 1.600–1.900 (data not shown).

We identified 11 optochin-resistant VGS isolates (30.6%) at the genus level by the MALDI-TOF MS tool matched with the results from *recA* sequencing confirmation. *S. oralis* isolates were the most common matched isolates between MALDI-TOF MS and recA sequencing tools (six strains), followed by *S. mitis* and *S. peroris* (two strains each) and *S. infantis* (one strain). In this study, we observed that only one optochin-resistant VGS isolate was identified at the genus level by the MALDI-TOF MS tool (Table 1). After *recA* sequencing confirmation, 17 optochin-resistant VGS isolated at the genus level by the MALDI-TOF MS tool were identified as *S. infantis* (Table 2).

**Figure 1:**
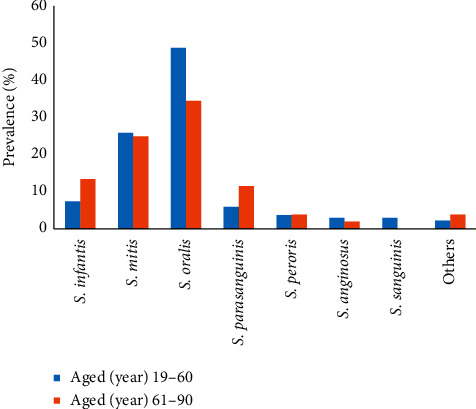
Prevalence of viridans group isolates isolated from adult patients with age 19 to 60 years (blue bar) and adult patients with age above 60 years (orange bar).

**Figure 2:**
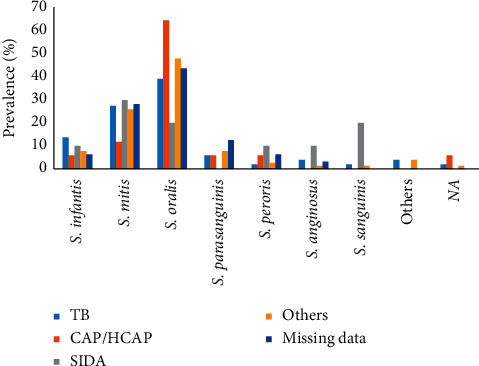
Prevalence of viridans group isolates isolated from adult patients with different clinical symptoms: tuberculosis (TB; blue bar), community-acquired pneumonia/healthcare-associated pneumonia (CAP/HCAP) (orange bar), SIDA/AIDS (gray bar), other symptoms (yellow bar), and missing data (blue dark bar).

In total, we identified that the optochin-resistant VGS species isolated from sputum samples were *S. oralis* (44.9%) followed by *S. mitis* (25.7%), *S. infantis* (9.1%), *S. parasanguinis* (7.5%), *S. peroris* (3.7%), *S. anginosus* (2.7%), *S. sanguinis* (2.1%), and others (2.7%). *S. oralis* isolates were found to be higher in patients with age between 19 and 60 years compared to patients aged above 60 years (Figure 1). Meanwhile, *S. infantis* and *S. parasanguinis* were more often isolated from older patients than young patients. We also observed that *S. oralis* were more often isolated from sputum specimens of adult patients with community-acquired pneumonia/healthcare-associated pneumonia (64.7%) and tuberculosis (39.2%) symptoms (Figure 2).

## 4. Discussion

In this study, we found that *S. oralis* and *S. mitis* were the major common optochin-resistant VGS isolates (70.6%) obtained from the sputum samples. The prevalence of *S. mitis* and *S. oralis* in this study was higher compared to other previous studies. Maeda et al. reported that the prevalence of *S. mitis* and *S. oralis* isolates from the sputum samples of adult patients with cystic fibrosis was 19% and 11%, respectively [14]. Meanwhile, the *S. anginosus* group (38.8%) and *S. mitis* (22.8%) group were the most common VGS species isolated from bloodstream infection detected by MALDI-TOF MS identification [15]. From oncologic patients, almost half of the VGS isolated from the blood culture was *S. mitis* isolates (46.5%) followed by *S. anginosus* (32.6%) and *S. sanguinis* (16.3%) by MALDI-TOF MS [6]. Oral streptococci isolates were reported as the most detected isolates from bronchoalveolar lavage fluid specimens obtained from pneumonia patients [16]. The oral streptococci isolates were all members of the *S. mutans* and *S. mitis* groups, the *S. salivarius* group, and the *S. anginosus* group except for *S. pneumoniae* [16]. *S. mitis* and *S. oralis* were significantly remaining species to be isolated from bloodstream isolates from neutropenic patients using the *sodA* gene detection [17].

In this study, we identified one isolate as *S. infantis* (2.6%) at the genus level. However, more *S. infantis* (9.1%) were identified from all optochin-resistant VGS isolates at the genus level by the MALDI-TOF MS tool after *recA* sequencing confirmation. Zbinden A et al. reported that the A 313-bp part of *recA* was selected on the basis of variability within the *S. mitis* group, showing <95.8% interspecies homology [13]. We found a mismatched pair of *S. pneumoniae* and *S. infantis* identified at the genus level by both MALDI-TOF MS and *recA* sequencing tools. This discrepancy was possibly due to high similarities in the molecular and proteomic profile of the mitis group including *S. mitis* and *S. oralis*, thus presenting a challenge to correctly identify species using DNA- or protein-based identification methods [2, 18]. The peak analysis and most updated Bruker database may improve the correct species identification [19]. In conclusion, the *S. oralis* and *S. mitis* were the predominant VGS isolates obtained from sputum of adult patients in Jakarta, Indonesia. MALDI-TOF MS showed potential for rapid identification to identify non-*Streptococcus pneumoniae* isolates. Although there were discrepancies in identifying isolates at the genus/species level, the performance could be improved by expanding its database.

## Figures and Tables

**Table 1:** MALDI-TOF MS identification for viridans group *Streptococcus* strains isolates from sputum samples of adult patients.

Species	MALDI-TOF MS identification	
	Secure to highly probable species^*∗*^, *n*(%)	Probable genus^#^, *n* (%)
*S. infantis*	0	1 (2.6)
*S. anginosus*	5 (3.4)	0
*S. mitis*	46 (31.1)	9 (23.1)
*S. oralis*	75 (50.7)	15 (38.5)
*S. parasanguinis*	14 (9.5)	1 (2.6)
*S. peroris*	3 (2.0)	8 (20.5)
*S. pseudopneumoniae*	1 (0.7)	0
*S. sanguinis*	4 (	0
*S. pneumoniae*	0	5 (12.8)

^*∗*^Score values higher than 1.99. ^#^Score values between 1.7 and 1.99.

**Table 2:** Comparison of samples identified by MALDI-TOF with score 1.700–1.999 and *recA* sequence analysis.

Isolate	Identification methods	
	MALDI-TOF MS (ID score value)	*recA* sequencing (similarity score, %)
RIN 002	*S. infantis* (1.942)	*S. infantis* (96)
RIN-176	*S. mitis* (1.837)	*S. infantis* (93)
RIN 289	*S. mitis* (1.881)	*S. infantis* (99)
RIN 106	*S. mitis* (1.902)	*S. infantis* (96)
RIN 230	*S. mitis* (1.903)	*S. mitis* (98)
RIN-267	*S. mitis* (1.908)	*S. mitis* (94)
RIN 241	*S. mitis* (1.918)	*S. oralis* (96)
RIN-155	*S. mitis* (1.931)	*S. infantis* (95)
RIN-206	*S. mitis* (1.940)	*S. infantis/oralis* (95)
RIN 248	*S. mitis* (1.993)	*S. infantis* (98)
RIN-327	*S. oralis* (1.75)	*S. infantis* (93)
RIN 312	*S. oralis* (1.797)	*S. infantis* (99)
RIN 096	*S. oralis* (1.833)	*S. infantis* (96)
RIN-112	*S. oralis* (1.834)	*S. infantis/oralis* (95)
RIN 215	*S. oralis* (1.860)	*S. infantis* (97)
RIN 335	*S. oralis* (1.887)	*S. infantis* (95)
RIN-221	*S. oralis* (1.898)	*S. oralis* (94)
RIN-114	*S. oralis* (1.899)	*S. oralis* (94)
RIN-089	*S. oralis* (1.900)	*S. oralis* (95)
RIN-132	*S. oralis* (1.917)	*S. peroris* (94)
RIN 129	*S. oralis* (1.943)	*S. oralis* (99)
RIN 083	*S. oralis* (1.945)	*S. oralis* (97)
RIN 208	*S. oralis* (1.947)	*S. pneumoniae* (97)
RIN 025	*S. oralis* (1.951)	ND
RIN 082	*S. oralis* (1.977)	*S. oralis* (97)
RIN-253	*S. parasanguinis* (1.845)	*Streptococcus* sp. i-G2 (96)
RIN-296	*S. peroris* (1.751)	*S. peroris* (94)
RIN-062	*S. peroris* (1.775)	*S. infantis* (93)
RIN-352	*S. peroris* (1.877)	*S. oralis* (93)
RIN-113	*S. peroris* (1.884)	*S. infantis* (95)
RIN 052	*S. peroris* (1.926)	*S. infantis* (99)
RIN-188	*S. peroris* (1.928)	*S. infantis* (94)
RIN 190	*S. peroris* (1.943)	*S. infantis* (97)
RIN-142	*S. peroris* (1.997)	*S. peroris* (95)
RIN 022	*S. pneumoniae* (1.834)	ND^*∗*^
RIN 345	*S. pneumoniae* (1.848)	*S. oralis* (96)
RIN 092	*S. pneumoniae* (1.865)	*S. infantis* (97)
RIN 320	*S. pneumoniae* (1.896)	ND
RIN-226	*S. pneumoniae* (1.899)	*S. peroris* (95)

^*∗*^ND = not done.

## Data Availability

The MALDI-TOF and recA sequencing data used to support the findings of this study are available from the corresponding author upon request.
